# Surgical resection for persistent seroma, following modified radical mastectomy

**DOI:** 10.1186/1477-7819-5-104

**Published:** 2007-09-23

**Authors:** Marek Stanczyk, Bartlomiej Grala, Tomasz Zwierowicz, Marek Maruszynski

**Affiliations:** 1 Department of General, Vascular and Oncologic Surgery of Military Health Service Institute, Warsaw, Poland; 2 Department of Pathology of Military Health Service Institute, Warsaw, Poland

## Abstract

**Background:**

Seroma formation following modified radical mastectomy with axillary lymph node dissection for breast cancer is a most common wound complication. In our experience seroma occurs in approximately 50% of patients undergoing mastectomy. Postmastectomy seromas usually vanishes within a few weeks after operation.

**Case presentation:**

In this report we present the case of a 73 year old woman who had undergone mastectomy with axillary lymph node dissection for breast cancer, complicated by lymphorrhea and formation fibrous encapsulated seroma resistant to conservative treatment which required surgical resection.

**Conclusion:**

We stand in opinion that in some cases of prolonged seromatous effusion with confirmed formation of thick walled reservoir the operation with resection and closure of supplying regional lymph vessels may be the best treatment, if possible preceded by arm lymphoscyntygraphy.

## Background

A seroma is a serous fluid collection which may develop in the space between the chest wall and skin flaps following mastectomy with axillary lymph node dissection for breast cancer. Seroma formation following mastectomy is a most common wound complication [1]. The incidence of seroma has been shown to correlate with patient's age, breast size and hypertension, presence of malignant nodes in the axilla, previous surgical biopsy and use of heparin [2-7]. Seroma formation may be also operator dependent and related to surgical techniques [8-10]. In our experience seroma occurs in approximately 50% of patients undergoing modified radical mastectomy for breast cancer. Although it usually vanishes within a few weeks, some patients may require repeated aspirations even for a period of months. In this report we present the rare case of the fibrous encapsulated seroma resistant to conservative treatment which required surgical resection.

## Case presentation

**Figure 1:**
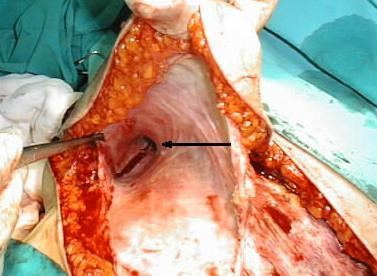
The spindle-like serous pouch located in the subcutaneus tissue on the greater pectoral muscle, arrow indicate vessel like structure penetrating deep in to posterior site of axilla.

**Figure 2:**
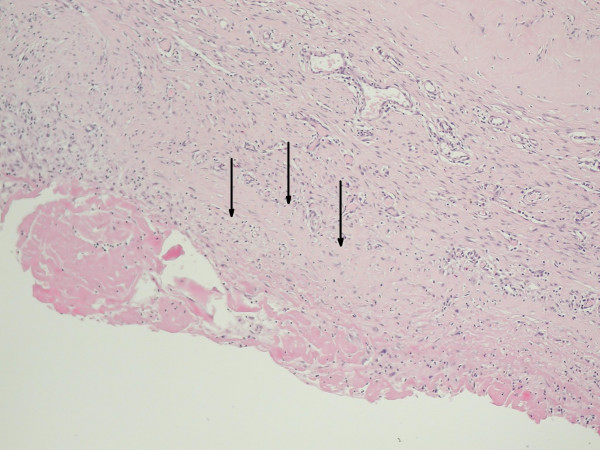
The seroma wall stained with hematoxylin/eosin under 100× magnification. Arrows indicate seroma capsule composed of fibrous tissue with eosinophilic hyaline degeneration of collagen and weak inflammatory, predominantly lymphocytic, infiltration. No epithelium was present on the inner surface of the seroma capsule.

A 73-year old woman was referred to our surgical department from a breast out-patient clinic, with a 1-year history of observation for subareolar tumor of right breast. Her medical history included arterial hypertension and osteoporosis. On clinical examination the tumor was palpable, approximately 2 cm in diameter, movable in relation to surrounding breast tissue and chest wall, axillary lymph nodes were not involved. Control mammography confirmed subareolar localization of the tumor, ranging from 11 to 15 mm in diameter. Biopsy revealed infiltrating ductal carcinoma, ER-75%, PR (-). A modified right radical mastectomy with level I and II lymph node dissection was performed without any complications. The wound was drained with two 16Fr, low pressure suction, silicone drains. One of the drains was placed in the axilla, a second was placed along the wound between skin flaps and greater pectoral muscle. Additionally external compressive dressing was placed on the wound site. Drainage volume declined from 220 ml on first post operative day to 50 ml on the sixth postoperative day and drainage was removed, total drained volume was 780 ml. Postoperative shoulder movements were not restricted and rehabilitation exercises started on the third postoperative day. The patient was discharged on the 7-th postoperative day. On control visit, 4 days after discharge, for skin sutures removal, patient showed with a palpable and symptomatic seroma in the wound site. She has undergone puncture with evacuation of 240 ml of clear serous fluid. Since then punctures were performed 2–3 times a week. Indication for puncture was symptomatic seroma causing pain and impairment of shoulder movement. Volume of clear serous fluid evacuated from wound site ranged from 120 ml to 260 ml per puncture. Additionally compressive dressings with elastic bandage were applied on the wound site. During the time of postoperative chemotherapy, the patient developed deep veins thrombosis of the lower extremities. Treatment with low molecular weight heparin (enoxaparin), increased the volume of evacuated fluid. On control USG there was no impairment of flow in axillary vessels, but USG of the wound showed spindle like container with thickened wall. Because volume of drainage didn't decline, after having finished chemotheraphy patient was qualified for operation as soon as symptoms of deep vein thrombosis diminished. Due to the large volume of drainage from the wound, we took into consideration not only inflammatory exudation as a cause, but also lymphatic origin of effusion. In this case surgical excision with closure of dead space may cause arm swelling in postoperative course. Because the patient didn't agree for lymphangiography, we decided to inject 2 ml of Patent Blue V dye subcutaneously in the right arm and fore arm 30 min before operation, in order to stain upper extremity lymph vessels confluence. The skin incision was done in the line of previous wound. The spindle-like serous pouch was located in the subcutaneous tissue on the greater pectoral muscle, with its lateral end forming vessel like structure and penetrating deep in to posterior site of axilla. (figure 1). After opening the reservoir, since there was no sign of stain in the lumen and containing fluid was clear, we assumed that there is only a little risk of arm swelling and lymph edema after resection of reservoir and closing of supporting vessel. After resection of reservoir, we visualized the route of the vessel supplying the reservoir by injection of Patent Blue V dye in to its lumen. The stained vessel branched on the lateral side of trunk and penetrated deep into intercostals of chest. All branches of stained vessel were closed separately. The wound was closed without suturing the dead space between the skin and greater pectoral muscle. The low pressure suction drain 16Fr was placed into axilla. Volume of drainage declined from 150 ml on first postoperative day to 50 ml on the fourth postoperative day, and drainage was removed. The patient was discharged on sixth postoperative day. She required two more punctures of serous fluid of total volume 140 ml, collected in the wound site. On the postoperative histological examination the seroma wall appeared to be composed of fibrous tissue with marked eosinophilic hyaline degeneration of collagen and weak predominantly lymphocyte infiltration. No epithelium was present on the inner surface of the seroma capsule. Despite accurate examination of the seroma wall no evidence of connection to lymphatic vessels was found, either macro- or microscopically (figure 2.)

## Discussion

A seroma is a serous fluid collection which develops in the space between the chest wall and skin flaps following mastectomy and axillary dissection. The Incidence of seroma formation following mastectomy may exceed 50% [1]. Although it usually vanishes within a few weeks, some patients may require repeated aspirations even for a period of months. Prolonged accumulation of postmastectomy seroma and repeated aspirations predisposes to sepsis, wound-related complications and may delay adjuvant therapy. There are many factors, which may increase or decrease seroma formation [2,3]. The incidence of seroma has been shown to correlate with patient's age, breast size and hypertension, presence of malignant nodes in the axilla, previous surgical biopsy and use of heparin [4-7]. Seroma formation may be also operator dependent and related to surgical techniques. Closure of dead space by suturing the wound flaps reduces the incidence of seroma formation, while the use of tissue glue to close the dead space remains controversial [8-10]. Mechanical pressure does not reduce the drainage of seroma [11]. Some evidence exists that tissue injury due to diathermy may be the cause of increased seroma formation [12]. The kind of drainage or no drainage and the timing of drain removal remain controversial. Closed suction drainage systems are preferable to open systems [13]. There is no advantage on amount or duration of drainage in use of single or multiple drains [14]. Comparison between low versus high vacuum drainage revealed no significant difference in the volume of fluid, duration of drainage or complication rate in studied groups of patients [15]. Some studies have advocated that the early removal of drains within the first week leads to increased seroma incidence, whereas others have shown that removal within five days has no influence [16]. It has been suggested prolonged drainage may perpetuate the volume drained, as drains can cause tissue inflammation. Leaving no drainage may lead to higher seroma formation rate and may be controversial but applicable to certain group of patients. Shortening of hospital stay may be achieved by early discharge with drain in situ. It was hypothesised that daily aspirations of seromas would keep the wound cavity dry and allow the wound flaps to adhere to the chest wall preventing accumulation of fluid, resulting in a more rapid resolution. This hypothesis was not proven, patients undergoing daily aspiration of seroma required significantly more aspirations without reducing the time from drain removal to final aspiration, therefore seromas should only be aspirated when symptomatic [17]. Delaying shoulder physiotherapy reduces drainage [18].

The origin of seroma is unclear. Studies on the composition of the fluid collected from post mastectomy drainage suggest its inflammatory origin while others hypothesized that seroma is most likely to originate from lymph [19,20]. In our case due to the large volume of seromatous fluid drained from the wound, both the inflammatory and lymphatic origin of effusion were taken in to consideration. If seroma formation or the subcutaneous collection of fluid was an inflammatory exudate, then careful surgical excision with closure of dead space may be effective in reducing this complication. But in case seromatous reservoir was connected and supplied by divided lymphatics collecting lymph from arm or trunk such excision would result in the development of both lymph edema and arm swelling. In the second case it would be more appropriate to find the lymphatic vessel supplying the reservoir and to perform vascular junction with axillary vein. Even though it is technically difficult and not commonly available in every hospital, lymphangiography of the right superior limb would be the method of choice for visualization of lymphatic drainage from the arm. The result of lymphangiography may be crucial for operative proceedings. In our case the patient did not agree for such examination. We decided to examine connection to lymphatics by injection of 2 ml of Patent Blue V subcutaneously in the arm and forearm 30 minutes prior to operation, speculating that lymphatic drainage from the upper limb if present, would color fluid in the serous reservoir. Although during resection we didn't observe change in drainage color, axillary end of the reservoir narrowed and formed a vessel which branched on the lateral side of trunk. That may suggest lymphatic compound of cistern formation. Those vessels were stained by injection of Patent Blue V dye and closed separately. On postoperative histological examination the connection of reservoir to lymphatic vessel was not proven. Even though there was no sign of vascular endothelium on the seroma wall and vessel supplying the superior end of seromatous reservoir, we considered that formation of such chronic seroma may have twofold etiology, both inflammatory and lymphatic. A similar case of fibrous encapsulated seroma following radical mastectomy, resistant to conservative treatment which finally required surgical resection was previously reported by Yoichi Matsui et all [21].

## Conclusion

We stand in opinion that in some cases of prolonged seromatous effusion with confirmed formation of thick walled reservoir the operation with resection and closure of supplying regional lymph vessels may be the best treatment, if possible preceded by lymphoscyntygraphy.

## Competing interests

The author(s) declare that they have no competing interests.

## Authors' contributions

MS operated the patient and wrote the manuscript

BG performed histological evaluation

TZ assisted during operation and corrected the manuscript

MM supervised the treatment and consulted the patient

All authors read and approved the final manuscript
